# Cell Adhesion Molecules Are Mediated by Photobiomodulation at 660 nm in Diabetic Wounded Fibroblast Cells

**DOI:** 10.3390/cells7040030

**Published:** 2018-04-16

**Authors:** Nicolette N. Houreld, Sandra M. Ayuk, Heidi Abrahamse

**Affiliations:** Laser Research Centre, Faculty of Health Sciences, University of Johannesburg, P.O. Box 17011, Doornfontein, Johannesburg 2028, South Africa; matabs63@yahoo.com (S.M.A.); habrahamse@uj.ac.za (H.A.)

**Keywords:** diabetes, extracellular matrix, fibroblasts, laser, photobiomodulation, wound healing

## Abstract

Diabetes affects extracellular matrix (ECM) metabolism, contributing to delayed wound healing and lower limb amputation. Application of light (photobiomodulation, PBM) has been shown to improve wound healing. This study aimed to evaluate the influence of PBM on cell adhesion molecules (CAMs) in diabetic wound healing. Isolated human skin fibroblasts were grouped into a diabetic wounded model. A diode laser at 660 nm with a fluence of 5 J/cm^2^ was used for irradiation and cells were analysed 48 h post-irradiation. Controls consisted of sham-irradiated (0 J/cm^2^) cells. Real-time reverse transcription (RT) quantitative polymerase chain reaction (qPCR) was used to determine the expression of CAM-related genes. Ten genes were up-regulated in diabetic wounded cells, while 25 genes were down-regulated. Genes were related to transmembrane molecules, cell–cell adhesion, and cell–matrix adhesion, and also included genes related to other CAM molecules. PBM at 660 nm modulated gene expression of various CAMs contributing to the increased healing seen in clinical practice. There is a need for new therapies to improve diabetic wound healing. The application of PBM alongside other clinical therapies may be very beneficial in treatment.

## 1. Introduction

### 1.1. Wound Healing and Diabetes

Wound healing is a physiological event critical to the continuity of life. It constitutes a complicated process whereby tissue injury results in a healing process involving various cell types including fibroblasts, cytokines, extracellular matrix (ECM) proteins, and certain growth factors to bring about tissue repair and restore integrity. Wound healing is as a result of four highly coordinated phases, namely haemostasis, inflammation, cell proliferation and remodelling. Fibroblasts are vital components of the ECM. They produce collagen that maintains cellular integrity, as well as activate the production of growth factors and other secretions to enhance wound repair. In conditions such as diabetes mellitus (DM), wounds are often stuck in the inflammatory phase, and there is increased inflammation and ECM degradation at the wound site due to decreased collagen production and increased proteolytic activity. There is also decreased production of growth factors and cytokines, with cells becoming unresponsive to growth factors, as well as reduced cell proliferation, and changes in gene expression [1,2,3,4,5]. The pathogenesis of DM is not properly comprehended; however, previous studies have shown that the production of several ECM factors are altered by hyperglycaemia. Culture conditions modulated expression of ECM proteins including cell adhesion molecules (CAMs) in vitro [6,7,8]. These pathological changes contribute to the development of chronic wounds, one of the most common complications associated with the disease which affects around 15% of patients [9]. Chronic foot ulcers often necessitate amputation, resulting in a decrease in the quality of life, as well as creating a socio-economic burden on the country and patients. Various therapeutic interventions, including photobiomodulation (PBM), have been used to improve wound healing in cases of diabetes and other stressed conditions.

### 1.2. Cell Adhesion Molecules

CAMs are binding proteins located on the surface of the cell and bind to other cells or the ECM. CAMs constitutes four superfamilies, namely the immunoglobulin superfamily (IgCAMs), integrins, selectins and cadherins, and play a vital role during wound healing. It is fundamental to regulate CAMs during tissue repair to enable cell migration, protein production and proliferation [10,11,12]. Cadherins and integrins are primarily cell surface transmembrane receptors. Their main function is to stimulate cell-cell and cell-matrix adhesion. They are also responsible for cell proliferation, differentiation, migration, survival and gene expression [13]. Integrins are heterodimeric transmembrane proteins that play important roles during developmental and pathological processes, including cell proliferation, differentiation, cell-cell attachment, adhesion and signal transduction between the cell and ECM [14]. Their adhesive nature allows them to bind to other ECM proteins such as vitronectin, fibronectin, laminin and collagen. Expressed integrins remain inactive and bind to their ligands only when activated. They mediate bi-directional signalling across the plasma membrane, inducing intracellular signals and are activated in three different states, low, basal and high, allowing them to change their expression in various receptors [15,16,17]. Various cell types express integrins, including fibroblasts [18].

Cadherins are calcium-independent adhesion molecules mainly involved in tissue and embryonic cell development. Their adhesive property is dependent on the ability of their intracellular domain to interact with cytoplasmic proteins. Cadherins are found at intercellular junctions that keep cells together, sense changes in mechanical tension and mechanotransducers and ensures adaptive support, and serve as a link between the ECM and the cytoskeleton to activate a large number of molecules and pathways [19,20]. Ig-CAMs express both heterophilic and homophylic binding of laminins, fibronectins, collagens and other cell surface proteins. They are particularly involved in cell-cell adhesion, embryogenesis and wound healing. Furthermore, they interact with integrin’s to enhance wound repair [21]. Selectins are up-regulated when stimulated. Fibroblasts are important in the secretion of soluble factors and synthesizing the ECM to regulate neovascularization [22,23]. Experiments on selectins have been shown to improve neovascularization, stromal cell derived factor (SDF)-α1 homing and wound healing [22]. E and P selectins also regulate inflammatory cell infiltration and improves wound healing.

### 1.3. Photobiomodulation (PBM) 

The application of PBM is a non-invasive therapeutic process for wound healing involving low energy power lasers or light emitting diodes (LEDs). Through several chemical, cellular and biological processes, PBM is capable of relieving pain and inflammation [24,25], as well as enhancing wound healing. PBM has been known for its biostimulatory effect in wound healing through various wavelengths, typically in the visible and near-infrared (NIR), and at low energy densities (fluencies). However, its mechanism of action is not fully understood. Currently, what is known, and the most accepted theory is that photons are absorbed by mitochondrial cytochrome C oxidase (Cox) which results in increased electron transfer, resulting in increased intracellular reactive oxygen species (ROS) and increased production of adenosine triphosphate (ATP). Nitric oxide (NO) is also photodissociated from Cox, which when bound to it inhibits Cox activity [26]. This brings about changes in cellular redox potential and pH levels, potassium and calcium ions, and cyclic adenosine monophosphate (cAMP) levels (derivative of ATP), which all play an important role in signal transduction, and induce several transcription factors [27]. These changes will bring about changes and stimulate cellular proliferation and migration, increased production and release of growth factors and cytokines, as well stimulation of ECM accumulation. Studies have shown that PBM modulates fibroblast cell proliferation and gene expression in wounded models at different wavelengths and fluences [28,29,30,31,32]. Investigations on diabetic wound healing involving humans, rats and other animal models has also shown positive effects [33,34,35,36,37,38,39,40,41]. Discovering and understanding the functions of CAMs in wound healing will allow for identification of mechanisms in medical conditions such as in chronic wounds [16]. Therefore, this study aimed to evaluate the influence of PBM on CAMs in diabetic wound healing.

## 2. Materials and Methods

### 2.1. Cell Isolation and Culure

Tissue was donated and collected from a consenting adult donor undergoing abdominoplasty (Linksfield, Sandringham, Johannesburg, South Africa). The isolation of cells from such tissue received ethical approval from the University of Johannesburg, Faculty Academic Ethics Committee (Clearance Reference Number: AEC05/01-2011). Briefly, adipose tissue was separated from the dermis with a sterile scalpel and the skin cut into 2–3 cm^2^ pieces. These smaller pieces of tissue were then incubated overnight at 37 °C, 5% CO_2_ in 10 mL of 0.25% Trypsin-1 mM Ethylenediaminetetraacetic acid (EDTA, ThermoFisher Scientific, Fairland, Johannesburg, South Africa, 25200-056). Trypsin was neutralised with an equal volume of media (Minimum Essential Medium, MEM, Sigma-Aldrich, Aston Manor, Johannesburg, South Africa, M7278) containing 10% Foetal Bovine Serum (FBS, Sigma-Aldrich, F9665). The dermis was then pulled away from and separated from the epidermis using a forcep. The dermis was then digested with 15 mL of 10 mg/mL collagenase-1 in Hanks Balanced Salt Solution (HBSS) with calcium (Ca^2+^) and magnesium (Mg^2+^) ions (Sigma-Aldrich, 55037C) and incubated for 40 min at 37 °C at 100 rpm (Labcon, Krugersdorp, Johannesburg, South Africa, shaker incubator, 3081u). Tissue was mechanically dissociated by pipetting several times (25 mL to 10 mL and then 5 mL disposable pipettes). Isolated cells were re-suspended in 20 mL complete MEM and cultured in T75 tissue culture flasks incubated overnight at 37 °C, 5% CO_2_. The media was selective for the growth of fibroblasts.

Isolated cells were cultured according to standard culture methods [42]. Briefly, cells were incubated at 37 °C, 5% CO_2_ in MEM supplemented with 10% FBS, 1 mM sodium pyruvate (ThermoFisher Scientific, Fairland, Johannesburg, South Africa, 11360-039), 2 mM l-glutamine (ThermoFisher Scientific, Gibco^®^, 25030-024), 0.1 mM non-essential amino acids (NEAA, ThermoFisher Scientific, Gibco^®^, 11140-035), 1% Penicillin-Streptomycin (ThermoFisher Scientific, Gibco^®^, 15140-122) and 0.2% Amphotericin-B (ThermoFisher Scientific, Gibco^®^, 15290-020) in T75/T175 culture flasks. Cells were detached with 1 mL/25 cm^2^ TrypLE™ Express (ThermoFisher Scientific, Gibco^®^, 12604-021). Cells between passage numbers 7 and 12 were used in experiments. A diabetic wounded model was achieved by continuously growing cells under hyperglycemic conditions; cells were cultured in complete media (basal glucose concentration of 5.6 mMol/L) containing an additional 17 mMol/L d-glucose [43,44]. Mimicking a diabetic condition using high glucose concentrations of 20–40 mM/L is not uncommon in inducing cellular changes in different cell types in vitro [45,46,47,48]. For experiments, 6 × 10^5^ cells in 3 mL complete media were seeded into 3.4 cm diameter tissue culture plates and cells allowed to adhere during overnight incubation. The following morning cells were rinsed with warm HBSS and 1 mL fresh media added to cultures. A wound was simulated in vitro via the scratch assay [49,50,51] under the same conditions, whereby a sterile 1 mL disposable pipette was used to scrape the confluent monolayer in a straight line, creating a cell-free zone in the center with cells either side of the ‘wound’ [44]. 

### 2.2. Laser Irradiation

Following the creation of a central scratch, cells were incubated for 30 min. A continuous wave diode laser emitting at a wavelength of 660 nm (Fremont, CA, USA, RGBlase, TECIRL-100G-650SMA) was used to irradiate cells (Table 1) in 3.4 cm diameter tissue culture dishes. This wavelength and laser parameters were chosen as they have previously shown stimulatory effects on WS1 cells [52,53]. Cells were irradiated via fibber optics in the dark from the top and at a distance that created the same spot size as the culture dish (7 cm). The temperature of the culture media was recorded every 2 min during irradiation to eliminate any effects produced by heat [28]. The duration of laser irradiation was determined by the output power (and hence output power density). Sham-irradiated (0 J/cm^2^) cells were used as controls.

### 2.3. PCR Array

Forty-eight hours post-irradiation, cells were detached and total RNA isolated using the RNeasy Mini Kit (Whitehead Scientific, Cape Town, South Africa, Qiagen, Hilden, Germany, 74104) and QIAshredder homogenisers performed on the QIAcube (Qiagen) as previously described [53]. RNA was quantified on the Qubit™ fluorometer (Invitrogen) using the Quant-iT™ RNA Assay kit (ThermoFisher Scientific, Invitrogen, Q32852), and purity determined spectrophotometrically at A260 nm/A280 nm. cDNA was reverse transcribed from 1 μg total RNA by means of the Quanti-Tect Reverse Transcription Kit (Whitehead Scientific, Qiagen, 205311). Briefly, following treatment with DNase, sample was incubated at 42 °C for 5 min. RT master mix was added and samples incubated at 42 °C for 15 min followed by 3 min incubation at 95 °C. cDNA was stored at −20 °C until ready for qPCR.

The Human Extracellular Matrix and Adhesion Molecules RT^2^ Profiler™ PCR Array (Whitehead Scientific, SABiosciences, Frederick, MD, USA, PAHS-013Z) was used to profile the expression of 84 genes important for cell-cell and cell-matrix interactions as previously described [28]. Of these 84 genes, 64 were CAMs (Table 2). Briefly, 111 μL cDNA was added to the master mix (containing ROX as a reference dye and SYBR green) and 25 μL was added to each well. Real-time qPCR was performed on the Stratagene MX3000p (Diagnostech, Dainfern Valley, Johannesburg, South Africa, Agilent Technologies, Waldbronn, Germany) using the following cycles: 1 cycle at 95 °C for 10 min; 40 cycles at 95 °C for 15 s and 60 °C for 1 min. A dissociation curve was performed at the end of the program to ensure the amplification of a single product (single peak at temperatures greater than 80 °C had to be obtained). Results were normalised against an average of all five housekeeping/reference genes and analysed on an excel based spreadsheet (Available from the SABiosciences website, http://www.sabiosciences.com). Threshold cycle (Ct) values greater than 35 were considered negative. Normalised expression levels from irradiated cells were calculated relative to non-irradiated control cells according to the 2^−ΔΔCt^ method and the genes were considered up- or down-regulated if the difference was >1 or <1, respectively.

### 2.4. Statistical Analysis

Experiments were repeated three times (*n* = 3). Results were normalised against an average of all five housekeeping/reference genes. The student *t*-test was performed by the SABiosciences Excel-based Data Analysis Template and reported as significant if *p* <0.05.

## 3. Results

**Figure 1:**
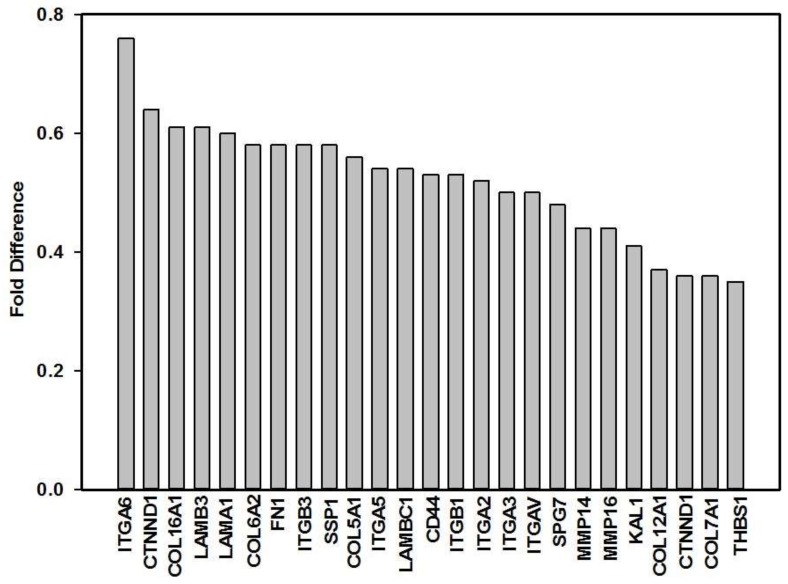
Significant down-regulation (fold difference <1) of genes related to CAMs in diabetic wounded cells irradiated with 660 nm at 5 J/cm^2^.

**Figure 2:**
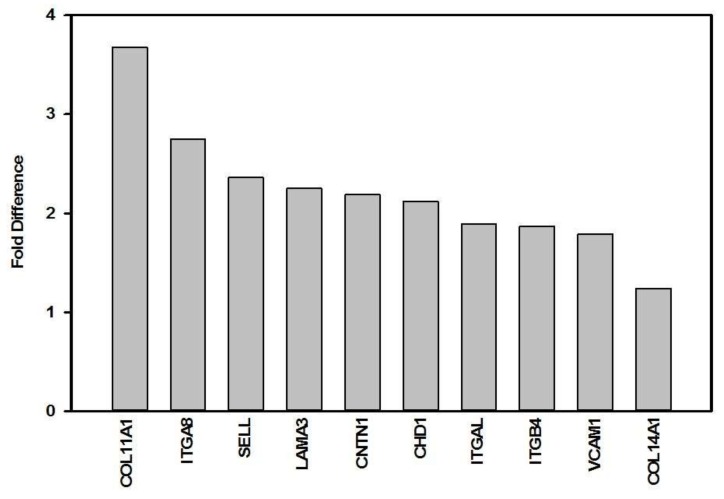
Significant up-regulation (fold difference >1) of genes related to CAMs in diabetic wounded cells irradiated with 660 nm at 5 J/cm^2^.

Forty-eight hours post-irradiation at a wavelength of 660 nm with 5 J/cm^2^, 64 genes related to CAMs was determined by real-time RT-qPCR in an in vitro diabetic wounded model (Table 3). Of the 64 genes, 25 were significantly down-regulated (Figure 1), while ten were significantly up-regulated (Figure 2). Eleven genes coding for transmembrane molecules were down-regulated (*CD44*, *ITGA2*, *ITGA3*, *ITGA5*, *ITGA6*, *ITGAV*, *ITGB1*, *ITGB3*, *MMP14*, *MMP16*, and *SPG7*), while five were up-regulated (*CDH1*, *ITGA8*, *ITGAL*, *ITGB4*, *SELL*, and *VCAM1*). Three genes coding for cell-cell adhesion molecules were down-regulated (*CD44*, *COL6A2*, and *CTNND1*), while five were up-regulated (*CDH1*, *COL11A1*, *COL14A1*, *ITGA8* and *VCAM1*). Nine genes coding for cell-matrix molecules were down-regulated (*CD44*, *ITGA2*, *ITGA3*, *ITGA5*, *ITGA6*, *ITGAV*, *ITGB1*, *ITGB3*, and *SSP1*), and three were up-regulated (*ITGA8*, *ITGAL*, and *ITGB4*). Genes related to other CAMs were also regulated by PBM, and 11 genes were down-regulated (*COL5A1*, *COL6A1*, *COL7A1*, *COL12A1*, *COL16A1*, *FN1*, *KAL1*, *LAMA1*, *LAMB3*, *LAMC1* and *THBS1*) and two were up-regulated (*CNTN1* and *LAMA3*).

## 4. Discussion

Wound healing is a carefully controlled and balanced process aimed at reversing the loss of structural integrity. CAMs play an important role during wound healing. Fibroblast cells play a major role in wound healing, and carry a variety of CAMs, and a deficiency in these CAMs may lead to delayed healing and ultimately chronic wounds [55]. Fibroblast cells produce many structural proteins important for wound healing, such as collagen. In addition, they also produce matrix metalloproteinases (MMPs), proteolytic enzymes which breakdown collagen. MMPs are necessary during the early stages of wound healing as they facilitate the movement of fibroblasts to the wound site. Later on in the wound healing stages, fibroblasts decrease their proteolytic activity and start producing structural proteins. When phases during wound healing do not progress, chronic wounds develop. 

The use of PBM both in vitro and in vivo has shown beneficial and simulative effects on wound healing [1,56,57,58,59,60]. Keshri et al. [56] studied the effect of PBM at 810 nm (22.6 J/cm^2^), using a pulsed (10 and 100 Hz, 50% duty cycle) diode laser, on full-thickness excision-type dermal wound healing in hydrocortisone-induced immunosuppressed rats. Their results showed accelerated healing through a decrease in inflammation (nuclear factor (NF)-kB, tumour necrosis factor (TNF)-α), and enhanced wound contraction (α-(SM) smooth muscle actin), cellular proliferation, ECM deposition, neovascularization (hypoxia inducible factor (HIF)-1α, and vascular endothelial growth factor, VEGF), and re-epithelialization. There was an up-regulation of the protein expression of fibroblast growth factor receptor-(FGFR)-1, fibronectin, heat shock protein (HSP)-90 and TGF-β2. Additionally, irradiation significantly increased cytochrome c oxidase (CCO, mitochondrial complex IV) activity and cellular adenosine triphosphate (ATP) content. Ayuk et al. [1] illustrated that diabetic wounded fibroblast cells irradiated at 660 nm with 5 J/cm^2^ presented with a significant increase in cell migration, viability, proliferation, and collagen content. In a similar study, Jere et al. [57] demonstrated that the increase observed in proliferation and migration may be due to activation of the JAK/STAT pathway in response to the binding of epidermal growth factor (EGF) to its receptor (EGFR). Tatmatsu Rocha et al. [58] demonstrated the effects of PBM at 904 nm in a diabetic wounded mouse model. Irradiated diabetic wounded mice displayed intense deposition and a more organized collagen matrix, and decreased concentration of nitrite, a marker of oxidative stress, and thiobarbituric acid (TBARS), a marker of lipid peroxidation. They showed that PBM was able to reduce nitrosative/oxidative stress in diabetic mice, thus accelerating wound healing. Beckmann et al. [59] conducted a systematic review on PBM (also referred to as low level laser therapy, or LLLT). They analysed 22 references; 8 in vitro studies, 6 animal studies, and 8 clinical trials. In vitro and animal studies provided proof of enhanced cellular migration, viability, and proliferation, rapid re-epithelization and reformed connective tissue, improved microcirculation, and anti-inflammatory effects. The clinical studies showed a potential benefit of PBM in the healing of diabetic ulcers, and stressed that better designed research trials are necessary. Ruh et al. [60] investigated the gene expression of inflammatory or reparative factors (interleukin-6, IL6; TNF; VEGF; and TGF) in pressure ulcers from eight patients which were irradiated at 660 nm with 2 J/cm^2^. Analysis of the lesions post-irradiation showed a 50% improvement in the size of granulation tissue, with a decrease in the gene expression in TNF-α, and an increase in VEFG and TGF-β. They concluded that PBM may be a beneficial complementary treatment for pressure ulcers.

CAMs play a vital role in wound healing, and is important in cellular proliferation, migration and the production of proteins [16]. During the initial phases of wound healing, fibrinogen, fibronectin, and vitronectin are produced to establish the provisional wound matrix of the blood clot. Other ECM proteins such as osteopontin, thrombospondins, secreted protein acidic and rich in cysteine (SPARC), tenascins, and collagens are released, and are only briefly present [16]. This study found a down-regulation in fibronectin-1, thrombospondin-1 (no effect on thrombospondin-2 and -3), CD44 (a glycoprotein which interacts with osteopontin), and collagen types -V, -VI, -VII, -XII, and -XVI in diabetic wounded fibroblast cells exposed to PBM at 660 nm with 5 J/cm^2^ and left to incubate for 48 h. No effect on the expression of tenascin C and vitronectin was observed. Tenascin-C holds both adhesive and anti-adhesive properties, depending on the cellular context. Keshri et al. [56] found an increase in the expression of fibronectin when using an 810 nm pulsed laser in an immunosuppressed rat wounded model. In this study, there was a significant up-regulation in the expression of collagen type -XI and -XIV. Both these collagens are involved in fibrillogenesis. Fibril formation during wound healing is primarily carried out by collagen type I and III. Previous studies in the same cells have shown an increase in collagen type I secretion, as well as its up-regulation in response to irradiation at 660 nm with 5 J/cm^2^ [1,61]. Carvalho et al. [62] and Colombo et al. [63] both found increased collagen and improved healing in wounded rat models in response to PBM. In this study, there was a significant down-regulation in proteases *MMP14* and *MMP16*, as well as *SPG7*, which codes for a mitochondrial metalloprotease and is necessary for intracellular motility and membrane trafficking. MMPs are secreted as inactive pro-proteins and require cleavage for activation. They are needed during tissue remodelling and maintaining the balance of the ECM. Often in the case of DM, MMP levels are elevated which contributes to the non-healing of chronic wounds [64]. Gharagozlian et al. [64] showed that PBM at 810 nm (1 or 3 J/cm^2^) reduced the gene expression of *MMP3*, *MMP9*, and *MMP13* in Achilles tendons of rats. Casalechi et al. [65] showed that a wavelength of 780 nm (7.5 J/cm^2^) modulated gene expression of *MMP1* and *MMP13* in Wistar rats. 

Integrins form a cell-surface receptor for collagen and laminin, and are the main mediators of cell attachment to the ECM [66]. This super-family consists of 24 varieties, all formed by one of 18 alpha subunits and one of 8 beta subunits. Extracellular ligand binding (such as fibronectin and laminin) promotes intracellular signalling. Integrins play a vital role in cell migration, and also allow cells to interact with the wound ECM. Several integrins are functionally stimulated or their expression is up-regulated in response to their contact with ECM molecules [16]. In this study, there was a significant down-regulation in a number of integrin alpha subunits: A2, which is expressed in the initial stages of wound healing and is involved in platelet adhesion; A3, which binds to members of the laminin family; A5, which associates with the beta 1 subunit to form a fibronectin receptor; A6, which associates with beta 1 or 4 subunits and interacts with members of the laminin family; and *ITGAV* which regulates angiogenesis. Integrin alpha 8 and integrin alpha L were both significantly up-regulated. The alpha 8 subunit regulates the recruitment of mesenchymal cells and mediates cell-cell interactions, while the alpha L chain associates with the beta 2 sub-chain (which was approaching significant up-regulation in this study) to form a receptor for lymphocytes. Members of the integrin beta subunits were also down-regulated: B1 and B3, both of which binds to alpha 5 subunit, which was also down-regulated. The gene coding for the integrin beta 4 subunit was up-regulated. This subunit acts as a receptor for the laminin family. In a study conducted by Giuliani and colleagues [67] the daily irradiation of mouse embryonic fibroblasts for 3 days to a wavelength of 670 nm (pulsed wave, 0.21 mW/cm^2^, 4.3 mJ/cm^2^) resulted in the up-regulation of *ITGA5*. This is in contract to our study, but may be due to the different irradiation protocols. 

Laminins are important in the formation and functioning of the basement membrane, and are vital to cell adhesion, differentiation, migration and signalling. Forty-eight hours post-irradiation at 660 nm, diabetic wounded cells showed down-regulation in the genes coding for the alpha 1, beta 3 and gamma 1 subunits (*LAMA1*, *LAMB3* and *LAMC1*, respectively). Genes coding for the alpha 3 (*LAMA3*) subunit was up-regulated. This gene is responsive to several growth factors, including keratinocyte growth factor, epidermal growth factor (EGF) and insulin-like growth factor (IGF). In a similar study, diabetic wounded fibroblast cells irradiated at the same parameters as in this study (660 nm; 5 J/cm^2^) showed increased secretion of EGF, and subsequent activation of its receptor, EGFR [57]. Giuliani and colleagues [67] found no effect on the gene regulation of *LAMA1* when they irradiated mouse embryonic fibroblasts (670 nm, 4.3 mJ/cm^2^). They also found no effect on cadherin 1. In contrast, this study found a significant up-regulation in *CDH1*, which codes for cadherin 1. Cadherins are involved in cell–cell adhesion and promotes a distinctive cytoskeletal structure which provides adhesive strength [68]. This study found a significant down-regulation in *CTNND1*, and up-regulation in *CTNND2*. These genes code for adhesive junction proteins of the catenin family. Catenin delta 2 protein is found to promote the disruption of E-cadherin adherens junctions which favors the spreading of cells [54]. This study also found an up-regulation in the gene which codes for contactin 1, *CNTN1*. This protein forms part of the immunoglobulin family, and functions as a cell-adhesion molecule. The gene encoding for another immunoglobulin family member, *VCAM1*, was also up-regulated. This gene codes for vascular cell adhesion molecule 1, and is a transmembrane molecule. The gene for another transmembrane molecule, *SELL*, was also up-regulated. This gene codes for selectin L. The *KAL1* (or *ANOS1*) gene was down-regulated in cells 48 h post-irradiation at 660 nm. The protein coded for by this gene, Kallmann syndrome 1 sequence, is believed to have anti-protease activity [54]. 

## 5. Conclusions

Normal wound healing processes are disrupted and hindered in DM, and the management of chronic diabetic ulcers remains a major global social and clinical challenge. Current therapies remain inadequate, with repeated failure and relapse. Several papers have shown the beneficial effects of PBM on diabetic wound healing, with favourable outcomes and no reported side-effects. This study revealed that laser irradiation at a wavelength of 660 nm and a fluence of 5 J/cm^2^ had an effect on a number of CAMs in a diabetic wounded fibroblast cell model. PBM influenced genes coding for proteins related to transmembrane molecules, cell-cell adhesion, cell-matrix adhesion, and a number of other CAMs. The difference seen in some of these studies may be due to different irradiation protocols and time post-irradiation, which would affect phase of healing, as well as cell types and models used. All of these need to be taken into consideration. Despite this, it cannot be denied that PBM has a profound effect on the ECM and CAMs, leading to increased healing. 

## Figures and Tables

**Table 1:** Laser parameters.

Parameters	
Wavelength	660 nm
Output power	92.8 mW
Spot size	9.1 cm^2^
Output power density	10.22 mW/cm^2^
Fluence	5 J/cm^2^
Duration of irradiation	8 min 9 s

**Table 2:** Functional CAM gene grouping.

Pathway	Gene
Transmembrane Molecules	*CD44*, *CDH1*, *HAS1*, *ICAM1*, *ITGA1*, *ITGA2*, *ITGA3*, *ITGA4*, *ITGA5*, *ITGA6*, *ITGA7*, *ITGA8*, *ITGAL*, *ITGAM*, *ITGAV*, *ITGB1*, *ITGB2*, *ITGB3*, *ITGB4*, *ITGB5*, *MMP14*, *MMP15*, *MMP16*, *NCAM1*, *PECAM1*, *SELE*, *SELL*, *SELP*, *SGCE*, *SPG7*, *VCAM1*
Cell-Cell Adhesion	*CD44*, *CDH1*, *COL6A2*, *COL11A1*, *COL14A1*, *CTNND1*, *ICAM1*, *ITGA8*, *VCAM1*
Cell-Matrix Adhesion	*ADAMTS13*, *CD44*, *ITGA1*, *ITGA2*, *ITGA3*, *ITGA4*, *ITGA5*, *ITGA6*, *ITGA7*, *ITGA8*, *ITGAL*, *ITGAM*, *ITGAV*, *ITGB1*, *ITGB2*, *ITGB3*, *ITGB4*, *ITGB5*, *SGCE*, *SPP1*, *THBS3*
Other Adhesion Molecules	*CLEC3B*, *CNTN1*, *COL5A1*, *COL6A1*, *COL7A1*, *COL8A1*, *COL12A1*, *COL15A1*, *COL16A1*, *CTGF*, *CTNNA1*, *CTNNB1*, *CTNND2*, *FN1*, *KAL1*, *LAMA1*, *LAMA2*, *LAMA3*, *LAMB1*, *LAMB3*, *LAMC1*, *THBS1*, *THBS2*, *TNC*, *VCAN*, *VTN*
Housekeeping genes	*B2M*, *HPRT1*, *RPL13A*, *GAPDH*, *ACTB*

**Table 3:** Gene expression profile of CAMs in diabetic wounded human skin fibroblast cells irradiated at 660 nm with 5 J/cm^2^. * *p* ≤ 0.05; ** *p* ≤ 0.01; *** *p* ≤ 0.001.

Gene Symbol	Description	Gene ID	Fold Difference ^1^	*p*-Value	Gene Function [54]
*ADAMTS13*	ADAM metallopeptidase with thrombospondin type 1 motif, 13	11093	0.91	0.589	The enzyme encoded by this gene specifically cleaves von Willebrand Factor (vWF).
*CD44*	CD44 molecule (Indian blood group)	960	0.53	0.049 *	The protein encoded by this gene is a cell-surface glycoprotein involved in cell-cell interactions, cell adhesion and migration. It is a receptor for hyaluronic acid (HA) and can also interact with other ligands, such as osteopontin, collagens, and matrix metalloproteinases (MMPs).
*CDH1*	Cadherin 1, type 1, E-cadherin	999	2.12	0.050 *	This gene encodes a classical cadherin of the cadherin superfamily, and is a cell-cell adhesion protein.
*CTNNA1*	Catenin (cadherin-associated protein), alpha 1, 102 kDa	1495	0.83	0.340	This gene encodes a member of the catenin family of proteins that play an important role in cell adhesion process by connecting cadherins located on the plasma membrane to the actin filaments inside the cell.
*CTNNB1*	Catenin (cadherin-associated protein), beta 1, 88 kDa	1499	0.64	0.084	The protein encoded by this gene is part of a complex of proteins that constitute adherens junctions, which are necessary for the creation and maintenance of epithelial cell layers by regulating cell growth and adhesion between cells. They also anchor the actin cytoskeleton and may be responsible for transmitting the contact inhibition signal that causes cells to stop dividing once the epithelial sheet is complete.
*CTNND1*	Catenin (cadherin-associated protein), delta 1	1500	0.36	0.005 **	This gene encodes a member of the Armadillo protein family, which function in adhesion between cells and signal transduction.
*CTNND2*	Catenin (cadherin-associated protein), delta 2 (neural plakophilin-related arm-repeat protein)	1501	2.72	0.052	This gene encodes an adhesive junction associated protein of the armadillo/beta-catenin superfamily. The protein also promotes the disruption of E-cadherin based adherens junction to favour cell spreading upon stimulation by hepatocyte growth factor.
*CLEC3B*	C-type lectin domain family 3, member B	7123	1.14	0.749	This gene codes for the protein tetranectin which may be involved in the packaging of molecules destined for exocytosis.
*CNTN1*	Contactin 1	1272	2.19	0.041 *	The protein encoded by this gene is a member of the immunoglobulin superfamily. It is a glycosyl-phosphatidylinositol (GPI)-anchored neuronal membrane protein that functions as a cell adhesion molecule.
*CTGF*	Connective tissue growth factor	1490	1.10	0.521	The protein encoded by this gene is a mitogen that is secreted by vascular endothelial cells. The encoded protein plays a role in chondrocyte proliferation and differentiation, cell adhesion in many cell types, and is related to platelet-derived growth factor.
*COL5A1*	Collagen, type V, alpha 1	1289	0.56	0.036 *	This gene encodes an alpha chain for one of the low abundance fibrillar collagens. Type V collagen is found in tissues containing type I collagen and appears to regulate the assembly of heterotypic fibbers composed of both type I and type V collagen. This gene product is closely related to type XI collagen and it is possible that the collagen chains of types V and XI constitute a single collagen type with tissue-specific chain combinations.
*COL6A1*	Collagen, type VI, alpha 1	1291	0.64	0.015 *	The basic structural unit of collagen VI is a heterotrimer of the alpha1(VI), alpha2(VI), and alpha3(VI) chains. The protein encoded by this gene is the alpha 1 subunit of type VI collagen (alpha1(VI) chain). Collagen VI is a major structural component of microfibrils.
*COL6A2*	Collagen, type VI, alpha 2	1292	0.58	0.014 *	The protein encoded by this gene is the alpha 2 subunit of type VI collagen (alpha2(VI) chain). Type VI collagen is a beaded filament collagen found in most connective tissues. The product of this gene contains several domains similar to von Willebrand Factor type A domains. These domains have been shown to bind ECM proteins, which explains the importance of this collagen in organizing matrix components.
*COL7A1*	Collagen, type VII, alpha 1	1294	0.36	0.017 *	This gene encodes the alpha 1 subunit of type VII collagen. Type VII collagen fibril, composed of three identical alpha collagen chains, is restricted to the basement zone and functions as an anchoring fibril between the external epithelia and the underlying stroma.
*COL8A1*	Collagen, type VIII, alpha 1	1295	0.71	0.271	This gene encodes the alpha 1 subunit (one of two alpha chains) of type VIII collagen. The gene product is a short chain collagen and a major component of the basement membrane of the corneal endothelium.
*COL11A1*	Collagen, type XI, alpha 1	1301	3.68	0.002 **	This gene encodes the alpha 1 subunit (one of two chains) of type XI collagen, a minor fibrillar collagen.
*COL12A1*	Collagen, type XII, alpha 1	1303	0.37	0.003 **	This gene encodes the alpha 1 subunit of type XII collagen, a member of the FACIT (fibril-associated collagens with interrupted triple helices) collagen family. Type XII collagen is a homotrimer found in association with type I collagen, an association that is thought to modify the interactions between collagen I fibrils and the surrounding matrix.
*COL14A1*	Collagen, type XIV, alpha 1	7373	1.24	0.023 *	This gene encodes the alpha 1 subunit of type XIV collagen, a member of the FACIT collagen family. Type XIV collagen interacts with the fibril surface and is involved in the regulation of fibrillogenesis.
*COL15A1*	Collagen, type XV, alpha 1	1306	0.67	0.082	This gene encodes the alpha 1 subunit of type XV collagen, a member of the FACIT collagen family. Type XV collagen has a wide tissue distribution but the strongest expression is localized to basement membrane zones so it may function to adhere basement membranes to underlying connective tissue stroma.
*COL16A1*	Collagen, type XVI, alpha 1	1307	0.61	0.001 **	This gene encodes the alpha 1 subunit of type XVI collagen, a member of the FACIT collagen family. Members of this collagen family are found in association with fibril-forming collagens such as type I and II, and serve to maintain the integrity of the ECM.
*FN1*	Fibronectin 1	2335	0.58	0.008 **	This gene encodes fibronectin, a glycoprotein present in a dimeric or multimeric form at the cell surface and in ECM. The encoded preproprotein is proteolytically processed to generate the mature protein. Fibronectin is involved in cell adhesion and migration processes including wound healing.
*HAS1*	Hyaluronan synthase 1	3036	0.87	0.544	Hyaluronan or hyaluronic acid (HA) is a polysaccharide and is a constituent of the ECM. It serves a variety of functions, including space filling, lubrication of joints, and provision of a matrix through which cells can migrate. HA is actively produced during wound healing and tissue repair to provide a framework for ingrowth of blood vessels and fibroblasts. HA is synthesized by membrane-bound synthase at the inner surface of the plasma membrane.
*ICAM1*	Intercellular adhesion molecule 1	3383	0.97	0.843	This gene encodes a cell surface glycoprotein which is typically expressed on endothelial cells and cells of the immune system. It binds to integrins of type CD11a/CD18, or CD11b/CD18.
*ITGA1*	Integrin, alpha 1	3672	0.97	0.721	This gene encodes the alpha 1 subunit of integrin receptors. This protein heterodimerizes with the beta 1 subunit to form a cell-surface receptor for collagen and laminin. The heterodimeric receptor is involved in cell-cell adhesion and may play a role in inflammation and fibrosis. The alpha 1 subunit contains an inserted (I) von Willebrand factor type I domain which is thought to be involved in collagen binding.
*ITGA2*	Integrin, alpha 2 (CD49B, alpha 2 subunit of VLA-2 receptor)	3673	0.52	0.015 *	This gene encodes the alpha 2 subunit of a transmembrane receptor for collagens and related proteins. The encoded protein forms a heterodimer with a beta subunit and mediates the adhesion of platelets and other cell types to the ECM.
*ITGA3*	Integrin, alpha 3 (antigen CD49C, alpha 3 subunit of VLA-3 receptor)	3675	0.50	0.010 **	This gene encodes the alpha 3 subunit of integrin. Integrins are heterodimeric integral membrane proteins that function in cell surface adhesion, cytoskeletal rearrangement and cell signalling. This subunit joins with a beta 1 subunit to form an integrin that interacts with ECM proteins, including members of the laminin family.
*ITGA4*	Integrin, alpha 4 (antigen CD49D, alpha 4 subunit of VLA-4 receptor)	3676	0.86	0.459	This gene encodes for the alpha 4 subunit of the integrin alpha chain family. This subunit associates with a beta 1 or beta 7 subunit to form an integrin that may play a role in cell motility and migration.
*ITGA5*	Integrin, alpha 5 (fibronectin receptor, alpha polypeptide)	3678	0.54	0.004 **	This gene encodes for the alpha 5 subunit of the integrin alpha chain family. This subunit associates with the beta 1 subunit to form a fibronectin receptor.
*ITGA6*	Integrin, alpha 6	3655	0.76	0.012 *	This gene encodes for the alpha 6 subunit of the integrin alpha chain family. This subunit may associate with a beta 1 or beta 4 subunit to form an integrin that interacts with ECM proteins including members of the laminin family.
*ITGA7*	Integrin, alpha 7	3679	1.11	0.513	This gene encodes for the alpha 7 subunit of the integrin alpha chain family. This protein functions as a receptor for the basement membrane protein laminin-1. It is mainly expressed in skeletal and cardiac muscles and may be involved in differentiation and migration processes during myogenesis.
*ITGA8*	Integrin, alpha 8	8516	2.75	0.050 *	This gene encodes for the alpha 8 subunit of the heterodimeric integrin alpha8beta1 protein. The encoded protein is a single-pass type 1 membrane protein. This gene regulates the recruitment of mesenchymal cells into epithelial structures, mediates cell-cell interactions, and regulates neurite outgrowth of sensory and motor neurons. The integrin alpha8beta1 protein plays an important role in wound-healing and organogenesis.
*ITGAL*	Integrin, alpha L (antigen CD11A (p180), lymphocyte function-associated antigen 1; alpha polypeptide)	3683	1.89	0.003 **	This gene encodes the integrin alpha L chain. This I-domain containing alpha integrin combines with the beta 2 chain (ITGB2) to form the integrin lymphocyte function-associated antigen-1 (LFA-1). LFA-1 plays a central role in leukocyte intercellular adhesion through interactions with its ligands, ICAMs 1-3, and also functions in lymphocyte costimulatory signalling.
*ITGAM*	Integrin, alpha M (complement component 3 receptor 3 subunit)	3684	1.77	0.065	This gene encodes the integrin alpha M chain. This I-domain containing alpha integrin combines with the beta 2 chain (ITGB2) to form a leukocyte-specific integrin known as macrophage receptor 1 (‘Mac-1’), or inactivated-C3b (iC3b) receptor 3 (‘CR3’). The alpha M beta 2 integrin is important in the adherence of neutrophils and monocytes to stimulated endothelium, and also in the phagocytosis of complement coated particles.
*ITGAV*	Integrin, alpha V (vitronectin receptor, alpha polypeptide, antigen CD51)	3685	0.50	0.002 **	The product of this gene belongs to the integrin alpha chain family. This subunit associates with beta 1, beta 3, beta 5, beta 6 and beta 8 subunits. The heterodimer consisting of alpha V and beta 3 subunits is also known as the vitronectin receptor. This integrin may regulate angiogenesis.
*ITGB1*	Integrin, beta 1 (fibronectin receptor, beta polypeptide, antigen CD29 includes MDF2, MSK12)	3688	0.53	0.005 **	The product of this gene belongs to the integrin beta chain family; integrin beta chains combine with multiple different alpha chains to form different integrin heterodimers. The ITGB1 protein product is the integrin beta chain beta 1. Integrin family members are noncovalently associated transmembrane glycoprotein receptors involved in cell adhesion and recognition in a variety of processes including embryogenesis, hemostasis, tissue repair, and immune response.
*ITGB2*	Integrin, beta 2 (complement component 3 receptor 3 and 4 subunit)	3689	1.58	0.083	The ITGB2 protein product is the integrin beta chain beta 2. The encoded protein plays an important role in immune response.
*ITGB3*	Integrin, beta 3 (platelet glycoprotein IIIa, antigen CD61)	3690	0.58	0.006 **	The ITGB3 protein product is the integrin beta chain beta 3. Integrin beta 3 is found along with the alpha IIb chain in platelets.
*ITGB4*	Integrin, beta 4	3691	1.87	0.041 *	This gene encodes the integrin beta 4 subunit, a receptor for the laminins.
*ITGB5*	Integrin, beta 5	3693	0.97	0.879	This gene encodes the integrin subunit beta 5, and is involved in adhesion to vitronectin.
*KAL1*	Kallmann syndrome 1 sequence	3730	0.41	0.001 ***	Also known as the ANOS1 gene. The protein encoded for by this gene is a cell surface protein, which is N-glycosylated and may have anti-protease activity.
*LAMA1*	Laminin, alpha 1	284217	0.60	0.014 *	This gene encodes one of the alpha 1 subunits of laminin. Laminins are a family of ECM glycoproteins that have a heterotrimeric structure consisting of an alpha, beta and gamma chain which are bound to each other by disulphide bonds into a cross-shaped molecule. These proteins make up a major component of the basement membrane and have been implicated in a variety of biological processes including cell adhesion, differentiation, migration, and signalling.
*LAMA2*	Laminin, alpha 2	3908	1.17	0.396	This gene encodes the alpha 2 chain, which constitutes one of the subunits of laminin 2 (merosin) and laminin 4 (s-merosin).
*LAMA3*	Laminin, alpha 3	3909	2.25	0.031 *	This gene encodes the alpha 3 chain of laminins. Laminins are essential for formation and function of the basement membrane. This gene is responsive to several epithelial-mesenchymal regulators including keratinocyte growth factor, epidermal growth factor and insulin-like growth factor.
*LAMB1*	Laminin, beta 1	3912	0.95	0.609	This gene encodes the beta chain isoform laminin, beta 1.
*LAMB3*	Laminin, beta 3	3914	0.61	0.010 **	The product encoded by this gene is a beta subunit laminin, which together with an alpha and a gamma subunit, forms laminin-5
*LAMC1*	Laminin, gamma 1 (formerly LAMB2)	3915	0.54	0.008 **	This gene encodes the gamma chain isoform laminin, gamma 1. Embryos of transgenic mice in which both alleles of the gamma 1 chain gene were inactivated by homologous recombination, lacked basement membranes, indicating that laminin, gamma 1 chain is necessary for laminin heterotrimer assembly.
*MMP14*	Matrix metallopeptidase 14 (membrane-inserted)	4323	0.44	0.016 *	Proteins of the matrix metalloproteinase (MMP) family are involved in the breakdown of ECM in normal physiological processes, such as tissue remodelling. The protein encoded by this gene is a member of the membrane-type MMP (MT-MMP) subfamily; each member of this subfamily contains a potential transmembrane domain suggesting that these proteins are expressed at the cell surface rather than secreted.
*MMP15*	Matrix metallopeptidase 15 (membrane-inserted)	4324	1.24	0.157	This gene encodes a member of the peptidase M10 family and membrane-type subfamily of MMPs.
*MMP16*	Matrix metallopeptidase 16 (membrane-inserted)	4325	0.44	0.003 **	Most MMP’s are secreted as inactive pro-proteins which are activated when cleaved by extracellular proteinases. The protein encoded by this gene is a member of the MT-MMP subfamily and activates MMP2 by cleavage.
*NCAM1*	Neural cell adhesion molecule 1	4684	0.97	0.811	This gene encodes a cell adhesion protein which is a member of the immunoglobulin superfamily. The encoded protein is involved in cell-to-cell interactions as well as cell-matrix interactions during development and differentiation. The encoded protein has been shown to be involved in development of the nervous system, and for cells involved in the expansion of T cells and dendritic cells which play an important role in immune surveillance.
*PECAM1*	Platelet/endothelial cell adhesion molecule	5175	3.66	0.213	The protein encoded by this gene is found on the surface of platelets, monocytes, neutrophils, and some types of T-cells, and makes up a large portion of endothelial cell intercellular junctions. The encoded protein is a member of the immunoglobulin superfamily and is likely involved in leukocyte migration, angiogenesis, and integrin activation.
*SELE*	Selectin E	6401	1.50	0.078	The protein encoded by this gene is part of the selectin family of cell adhesion molecules, and is found in cytokine-stimulated endothelial cells and is thought to be responsible for the accumulation of blood leukocytes at sites of inflammation by mediating the adhesion of cells to the vascular lining.
*SELL*	Selectin L	6402	2.36	0.013 *	This gene encodes a cell surface adhesion molecule that belongs to a family of adhesion/homing receptors.
*SELP*	Selectin P (granule membrane protein 140 kDa, antigen CD62)	6403	1.90	0.249	This protein redistributes to the plasma membrane during platelet activation and degranulation and mediates the interaction of activated endothelial cells or platelets with leukocytes.
*SGCE*	Sarcoglycan, epsilon	8910	0.93	0.657	This gene encodes the epsilon member of the sarcoglycan family. Sarcoglycans are transmembrane proteins that are components of the dystrophin-glycoprotein complex, which link the actin cytoskeleton to the ECM.
*SPG7*	Spastic paraplegia 7 (pure and complicated autosomal recessive)	6687	0.48	0.005 **	This gene encodes a mitochondrial metalloprotease protein that is a member of the AAA family. Members of this protein family share an ATPase domain and have roles in diverse cellular processes including membrane trafficking, intracellular motility, organelle biogenesis, protein folding, and proteolysis.
*SPP1*	Secreted phosphoprotein 1	6696	0.58	0.009 **	The protein encoded by this gene is involved in the attachment of osteoclasts to the mineralized bone matrix. This protein is also a cytokine that upregulates expression of interferon-gamma and interleukin-12.
*THBS1*	Thrombospondin 1	7057	0.35	0.001 ***	The protein encoded by this gene is a subunit of a disulphide-linked homotrimeric protein, and belongs to the thrombospondin family. This protein is an adhesive glycoprotein that mediates cell-to-cell and cell-to-matrix interactions. This protein can bind to fibrinogen, fibronectin, laminin, type V collagen and integrins alpha-V/beta-1.
*THBS2*	Thrombospondin 2	7058	0.81	0.088	The protein encoded by this gene belongs to the thrombo-spondin family.
*THBS3*	Thrombospondin 3	7059	1.03	0.794	The protein encoded by this gene belongs to the thrombo-spondin family.
*TNC*	Tenascin C	3371	1.18	0.492	This gene encodes for an ECM protein.
*VCAM1*	Vascular cell adhesion molecule 1	7412	1.79	0.002 **	This gene is a member of the Ig superfamily and encodes a cell surface sialoglycoprotein expressed by cytokine-activated endothelium. This type I membrane protein mediates leukocyte-endothelial cell adhesion and signal transduction.
*VCAN*	Versican	1462	0.96	0.775	This gene is a member of the aggrecan/versican proteoglycan family. The protein encoded is a large chondroitin sulphate proteoglycan and is a major component of the ECM. This protein is involved in cell adhesion, proliferation, migration and angiogenesis and plays a central role in tissue morphogenesis and maintenance.
*VTN*	Vitronectin	7448	1.20	0.240	The protein encoded by this gene is a member of the pexin family. It is found in serum and tissues and promotes cell adhesion and spreading, inhibits the membrane-damaging effect of the terminal cytolytic complement pathway, and binds to several serpin serine protease inhibitors.

^1^ A fold difference > 1 is considered as gene up-regulation, while a fold difference <1 is considered as gene down-regulation.
